# Characterization of Apoptosis-Related Oxidoreductases from *Neurospora crassa*

**DOI:** 10.1371/journal.pone.0034270

**Published:** 2012-03-28

**Authors:** Patrícia Carneiro, Margarida Duarte, Arnaldo Videira

**Affiliations:** 1 IBMC - Instituto de Biologia Molecular e Celular, Universidade do Porto, Porto, Portugal; 2 ICBAS - Instituto de Ciências Biomédicas de Abel Salazar, Universidade do Porto, Porto, Portugal; Universidade de Sao Paulo, Brazil

## Abstract

The genome from *Neurospora crassa* presented three open reading frames homologous to the genes coding for human AIF and AMID proteins, which are flavoproteins with oxidoreductase activities implicated in caspase-independent apoptosis. To investigate the role of these proteins, namely within the mitochondrial respiratory chain, we studied their cellular localization and characterized the respective null mutant strains. Efficiency of the respiratory chain was analyzed by oxygen consumption studies and supramolecular organization of the OXPHOS system was assessed through BN-PAGE analysis in the respective null mutant strains. The results demonstrate that, unlike in mammalian systems, disruption of AIF in Neurospora does not affect either complex I assembly or function. Furthermore, the mitochondrial respiratory chain complexes of the mutant strains display a similar supramolecular organization to that observed in the wild type strain. Further characterization revealed that *N. crassa* AIF appears localized to both the mitochondria and the cytoplasm, whereas AMID was found exclusively in the cytoplasm. AMID2 was detected in both mitochondria and cytoplasm of the amid mutant strain, but was barely discernible in wild type extracts, suggesting overlapping functions for the two proteins.

## Introduction

Mitochondria are regarded as the powerhouse of the cell producing the high levels of ATP required for life and death [1], [2]. Accordingly, mitochondrial dysfunction has been implicated in several human pathologies, including neurodegenerative disorders, cardiac dysfunction, diabetes and inflammatory diseases [3]–[6].

Mitochondria energy production is accomplished through a series of complexes present in the inner mitochondrial membrane that carry along oxidative phosphorylation and are thus known as OXPHOS complexes, of which type I NADH:ubiquinone oxidoreductase or complex I is the largest and most complex one [7], [8]. Complex I, composed of up to 45 subunits in mammals, couples electron transfer from NADH to ubiquinone with proton pumping across the inner mitochondrial membrane contributing a great deal to the proton motive force that will be used for ATP synthesis [9], [10]. In contrast, many organisms are known to contain highly branched mitochondrial respiratory chains encompassing type II NAD(P)H dehydrogenases that bypass complex I transferring electrons to ubiquinone in a rotenone-insensitive manner [11], [12].

Type II NAD(P)H dehydrogenases, also referred to as alternative dehydrogenases, are single polypeptides with FAD or FMN as prosthetic group that catalyze the oxidation of cytosolic or matrix NAD(P)H without proton translocation [13]. Alternative dehydrogenases, described in bacteria, protozoa, plants and fungi, have been proposed to be energy conservation bypasses and to provide plasticity under adverse environmental conditions, although their precise physiological relevance remains unclear [14], [15], [12].

Mitochondria are also known to contain other FAD-containing oxidoreductases, namely the apoptosis inducing factor (AIF) and the apoptosis-inducing factor-homologous mitochondrion-associated inducer of death (AMID) [16], [17], which have been assigned key roles in caspase-independent apoptosis. The human AIF has been identified as a mitochondrial mediator of caspase-independent apoptosis by translocating to the nucleus upon an apoptosis insult where it leads to chromatin condensation and large-scale DNA fragmentation [18], [19]. This ubiquitous 67 kDa protein encoded by a nuclear gene contains an amino-terminal mitochondrial localization signal (MLS), which is removed upon import into the mitochondria yielding the mature 62 kDa protein [20] and two nuclear localization signals (NLS) [21]. The C-terminal domain of AIF shares significant homology with oxidoreductases found in other vertebrates, plants and fungi, and indeed AIF has been characterized as a NAD(P)H oxidoreductase capable of generating superoxide anion [22]. Furthermore, AIF deficiency in mouse and human cells was found to hamper oxidative phosphorylation, specifically through an effect in the biogenesis and/or maintenance of respiratory complex I, and to a lower extent complex III, resulting in decreased respiratory activity [23]. However, AIF oxidoreductase activity is independent of its apoptogenic function and despite playing a role in OXPHOS, AIF has not been shown to be a part of the respiratory chain [22], [23].

AMID was characterized as an inducer of a novel caspase-independent apoptotic pathway [17]. Indeed, the human protein of about 40 kDa appears to be cytoplasmic and unlike AIF, AMID does not translocate to the nucleus during apoptosis despite inducing similar apoptotic effects on nuclear chromatin [24], [25], [17]. AMID has since been characterized as a novel p53-responsive death effector (PRG3) involved in the regulation of tumorigenesis upon findings of down regulation in a plethora of tumors [26]. With FAD-binding motifs in its N-terminal, AMID was found to be a flavoprotein with NADPH oxidoreductase activity independent of the apoptosis-inducing function and capable of binding DNA non-specifically [27].

In *Saccharomyces cerevisiae*, the internal alternative dehydrogenase NDI1 was identified as the yeast AMID homologue, capable of inducing apoptosis upon over expression in yeast, leading to typical apoptotic features like DNA fragmentation, chromatin condensation, increased ROS production and MOMP (mitochondrial outer membrane permeabilization) [28]. Additionally, an AIF orthologue was also shown to induce apoptosis in yeast, displaying typical nuclear translocation and death executing features upon apoptotic stimuli, although partially dependent on caspase activity [29]. In contrast, *Aspergillus nidulans* presents an AIF homologue highly expressed in farnesol-induced apoptosis that does not translocate to the nucleus upon cell death [30]. Interestingly, in the same study, the external NADH dehydrogenase NdeA was also shown to be up regulated upon farnesol (FOH) treatment. Dinamarco and colleagues have since shown that *A. nidulans* AifA plays a role in complex I function and that the mutant strain displays decreased resistance to FOH. Furthermore, the authors suggest that alternative dehydrogenases may play a specific role in FOH-induced cell death, possibly to overcome accumulation of ROS generated by complex I [31].

In *Neurospora crassa*, three genes were identified with recognized homology to AIF and AMID proteins, and the respective mutant strains have been shown to display different sensitivities to drug-induced apoptosis [32]. Specifically, our group has shown that whereas aif displays increased resistance to phytosphingosine (PHS), the amid strain is more sensitive. A putative anti-apoptotic role for AMID in *N. crassa* was suggested, despite the possibility that the AMID homologue in Neurospora could in fact be the other uncharacterized AMID-like protein [32].

Herein, we aimed to further characterize AIF and AMID proteins in *N. crassa*, specifically to understand their role in the fungal mitochondrial respiratory chain.

## Results

### *N. crassa* depicts three AIF-like proteins

**Figure 1:**
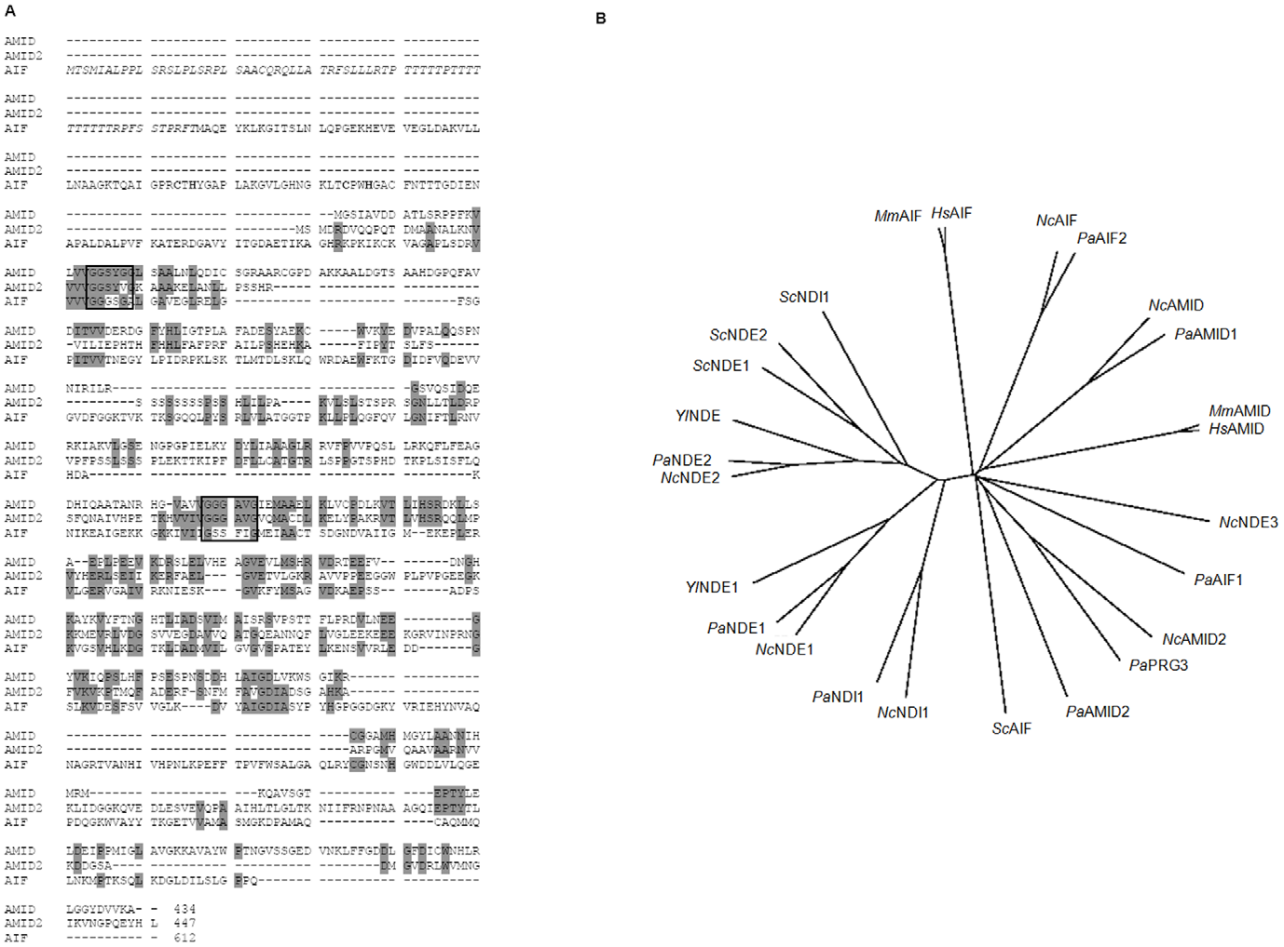
Apoptosis-related oxidoreductases from *N. crassa*. **(A) Sequence alignment of deduced proteins.** Identical amino acid residues present in at least two of the proteins are shown on a grey background. Amino acid regions containing the three G residues within conserved dinucleotide-binding motifs are depicted in a box. The residues involved in Rieske iron sulfur cluster coordination are shown in bold. Predicted mitochondrial pre-sequence is italicized. Gene locus numbers for AMID, AMID2 and AIF sequences are NCU06061.5, NCU12058.5 and NCU05850.5, respectively. **(B) Dendrogram of AIF-like oxidoreductases and type II NAD(P)H:quinone oxidoreductases.** Tree production was performed using Clustal X based on an amino acid sequence alignment of the proteins from eukaryotic organisms. The sequences used in neighbor-joining and respective abbreviations are listed in Table 3.

The genes coding for putative oxidoreductases belonging to the protein family of apoptosis-inducing factors AIF, AMID and AMID2 were identified in a BLAST search of the genomic database of the Neurospora Sequencing Project at the Whitehead Institute/MIT Center for Genome Research (www.genome.wi.mit.edu) using the previously characterized *S. cerevisiae* AIF protein sequence as query [29]. All three genes belong to linkage group VII, AIF is composed of 2 exons encoding a polypeptide of 612 amino acids, AMID is composed of five exons encoding a polypeptide of 434 aminoacids and AMID2 is composed of five exons encoding a polypeptide of 447 aminoacids. The predicted molecular masses of AIF, AMID and AMID2 are 65.5 kDa, 46.9 kDa and 48.3 kDa, respectively. Figure 1A depicts an alignment of the deduced primary sequences of all three proteins, revealing the conserved pyridine dinucleotide-binding motifs characteristic of NAD(P)H alternative dehydrogenases also present in AIF-like proteins [33]. Interestingly, Neurospora AIF presents residues involved in Rieske iron sulfur cluster coordination previously reported for other AIF-like proteins [34], [35] and, of the three proteins, it is the only one with a predicted mitochondrial targeting sequence (Fig. 1A). Likewise, the human AIF homologue depicts a MLS whereas the human AMID has been described as devoid of such signaling sequences [21], [25]. More so, the *N. crassa* proteins present recognized homology to human AIF suggesting that they represent the fungus AIF-like homologues. We performed an alignment of mammalian AIF and AMID sequences with alternative dehydrogenase and AIF-like sequences from fungi and the resulting dendogram revealed that the Neurospora alternative dehydrogenase NDE3 [36] appears to be closer to Neurospora AIF and AMID proteins than to the remaining alternative dehydrogenases (Fig. 1B). Furthermore, sequence alignment analysis revealed a higher degree of homology between NDE3 and AIF than between NDE3 and NDE2 Neurospora proteins (data not shown).

Overall, it appears that the *N. crassa* members of the AIF family share the general characteristics of the human homologues.

### AIF and AMID oxidoreductases are distributed between mitochondria and the cytoplasm

**Figure 2:**
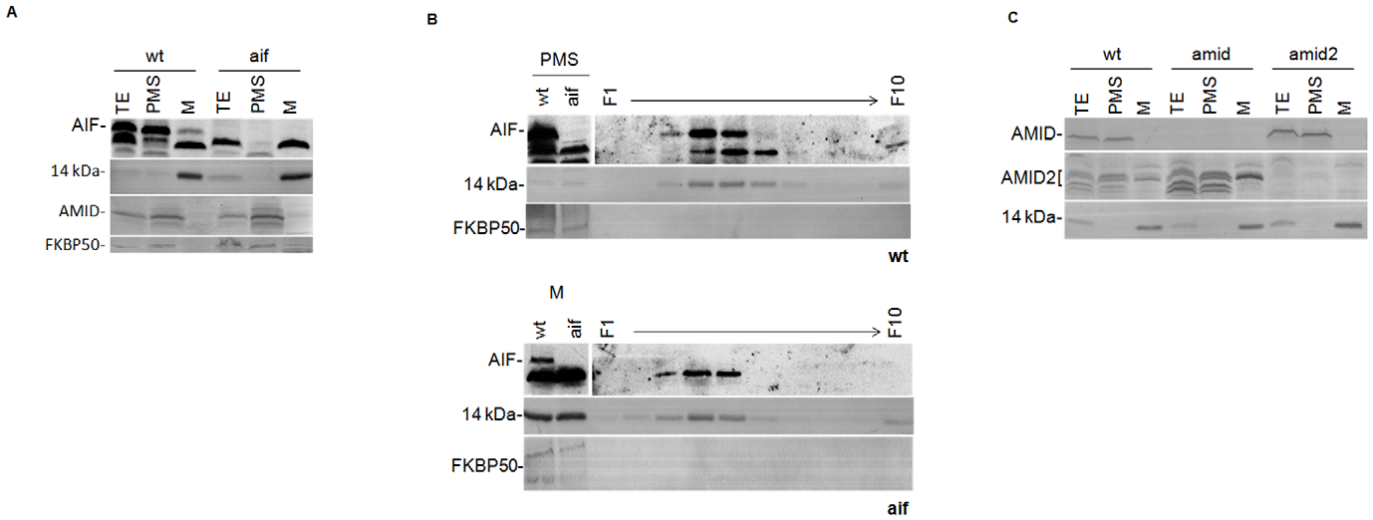
Localization of *N. crassa* AIF and AMID proteins. (A)Total cellular extracts (TE), cytoplasm (PMS) and mitochondria (M) from the wild type (wt) and aif were separated by SDS-PAGE and analyzed by Western blotting with antisera against AIF and AMID. (B) Mitochondria from wt and aif strains were fractionated upon centrifugation on a 30–60% (w/w) sucrose density gradient and analyzed by Western blotting with antibodies as indicated on the left. (C) As described above TE, PMS and M from wt and the indicated mutant strains were analyzed with antisera against AMID and AMID2. Antisera against FKBP50 [51] and the 14 kDa subunit of complex I [39] were used as purification controls.

We next determined the localization of the Neurospora AIF homologues through Western blot analysis of subcellular fractions isolated from cellular extracts grown under normal vegetative conditions. The respective deficient mutant strains, aif, amid and amid2, resulted from gene replacement by homologous recombination with the *hph* gene that confers resistance to hygromycin [37]. Overall, no significant growth or developmental phenotypes were observed in any of the strains as determined by phenotypic analyses of growth rates, asexual spore formation and sexual reproduction (data not shown). Specific polyclonal antisera against each protein were produced and subsequently used in Western blot analysis with the respective knock-out strains as negative controls. The amid2 mutant strain is a heterokaryon where three adjacent genes, including *amid-2*, were deleted due to inaccurate annotation. Thus, to ensure that FGSC#12511 conidia were deficient in *amid-2*, all experiments were performed in mycelium grown in the presence of hygromycin. As depicted in Fig. 2A, AIF was found mainly in the cytoplasm but it was also consistently detected in purified mitochondria, though in smaller amounts. Indeed, AIF was identified as a band with the expected molecular weight present in mitochondria (M) and cytoplasm (PMS-Post mitochondrial supernatant) from the wild type, which disappeared in extracts from the aif mutant. Additionally, the mitochondrial 14 kDa subunit of complex I, the cytoplasmic AMID (see Fig. 2C) and the nuclear FKBP50 proteins were used as controls. This was a surprising result given that AIF has been described as a mitochondrial protein that translocates to the nucleus upon apoptotic insults [18]. To confirm that AIF is indeed mitochondrial, crude mitochondria were separated on a linear sucrose density gradient (30 to 60%). The resulting fractions were analyzed by western blotting with antisera against AIF, the mitochondrial marker 14 kDa subunit of complex I [38] and the FKBP50 protein [39]. AIF was identified solely in the fractions containing mitochondria, as determined by the peaking of the 14 kDa protein (Fig. 2B). Although a small portion of the mitochondrial marker was detected on the top fraction we suggest that it results from organelle rupture during cell disruption. Antibodies against the cytoplasmic protein GAPDH and the ER protein FKBP22 were found not to peak with the mitochondrial marker, confirming the purity of the fractions (data not shown).

Interestingly, AMID2 was almost undetectable in wild type extracts of *N. crassa*, but was clearly visible in both the mitochondria and the cytoplasm of the Neurospora amid mutant, suggesting overlapping functions for AMID and AMID2 in the fungus (Fig. 2C). In contrast, AMID localized exclusively to the cytoplasm of *N. crassa*, in agreement with other reports suggesting a cytoplasmic localization for AMID proteins [25].

Thus, we conclude that AIF-like oxidoreductases in Neurospora are distributed in mitochondria and cytoplasm as reported for other organisms, although the double localization suggests a fungal-specific role for AIF.

### Disruption of AIF or AMID does not affect complex I activity

AIF is regarded as playing a key role in the mitochondrial respiratory chain, mainly in the assembly and function of complex I [23]. Thus, to assess the role of AIF and AMID in Neurospora mitochondria, we analyzed the efficiency of respiration by either mutant through oxygen consumption measurements (Table 1). The oxidation rates of matrix NADH (generated by pyruvate/malate) can be attributed to either complex I or to the internal alternative dehydrogenase NDI1, and are rotenone-sensitive or -insensitive, respectively. Accordingly, matrix NADH activities inhibited by rotenone in mitochondria from either mutant did not differ significantly from those of the wild type strain, suggesting that in contrast to the mammalian situation, deficiency of AIF in *N. crassa* does not affect complex I activity. In addition, our results suggest that AMID also does not affect complex I functioning, which is consistent with its cytoplasmic localization. The remaining rotenone-insensitive matrix NADH activity was also not significantly different between mutant and wild type strains (Table 1).

**Table 1:** Enzymatic activities of mitochondria from AIF-like mutant strains.

	wt	amid	aif
**NADH:HAR reductase** (arbitrary units)	0.59_±0.18_	0.41_±0.14_	0.63_±0.16_
**NADH oxidase** nmol O_2_/min/mg	95.1_±23.4_	77.0_±58.0_	52.2_±21.5_
**Malate:O_2_** nmolO_2_/min/mg (rotenone sensitive)	25.1_±9.0_	37.4_±16.1_	32.8_±9.3_
**Malate:O_2_** nmolO_2_/min/mg (rotenone insensitive)	19.2_±5.1_	12.0_±2.5_	11.2_±2.5_

All activities shown were completely inhibited by antimycin A in wild type and mutant strains. Their values represent the mean values ± S. D. obtained from at least three different mitochondrial preparations.

Moreover, the rates of oxygen consumption upon addition of exogenous NADH or NADPH were also comparable in the mutant and wild type strains, suggesting that deficiency of AIF or AMID does not hinder the activity of external NAD(P)H dehydrogenases (Table 1; data not shown). Interestingly, these results also provide evidence that mitochondrial AIF is not a member of the Neurospora respiratory chain and corroborates our report that the entry point of electrons into the respiratory chain is identified in the fungus [36].

**Figure 3:**
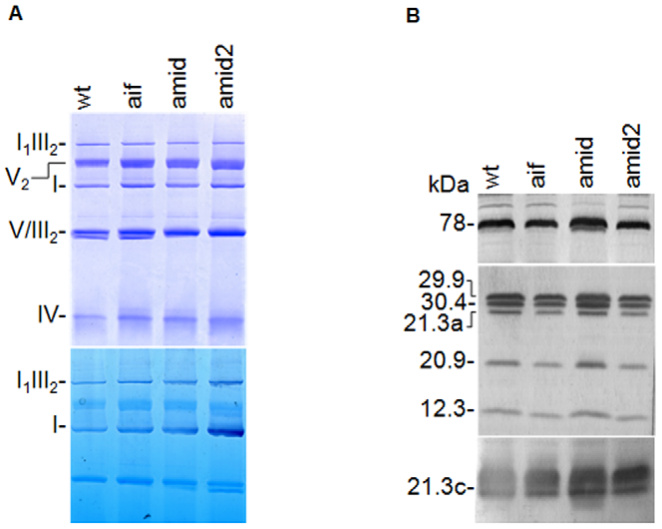
Analysis of mitochondrial OXPHOS complexes and complex I composition of *N. crassa* wild type and AIF-like mutants. Mitochondrial proteins from wild type, aif, amid and amid2 strains were resolved by BN-PAGE, stained with Coomassie blue (A – upper panel) or NADH/NBT activity (A – lower panel), and by SDS-PAGE immunoblot probed with antisera against the indicated subunits of complex I (B). OXPHOS complexes and supercomplexes are indicated on the left side of panel A [59], [60].

To further characterize the effect of disrupting AIF homologues in the Neurospora mitochondrial respiratory chain we evaluated the supramolecular organization of the OXPHOS system by electrophoretic analyses (Fig. 3A). Mitochondrial proteins from each strain were solubilized with the non-ionic detergent digitonin and resolved by BN-PAGE. We could detect the same respiratory complexes and supercomplexes upon Coomassie blue and in-gel NADH/NBT activity staining in all strains, indicating that AIF and AMID do not interfere with the assembly of respiratory chain complexes in *N. crassa*. Furthermore, we confirmed complex I integrity in aif and amid mutant strains by Western blot analyses with specific antisera against a series of complex I subunits (Fig. 3B).

Overall our results suggest that absence of AIF-like proteins in Neurospora mitochondria does not hamper complex I activity under normal vegetative conditions. More so, AIF or AMID, despite being recognized as oxidoreductases, do not appear to function in the mitochondrial respiratory chain in *N. crassa*, suggesting an independent yet uncharacterized oxidoreductase activity.

### Genetic interactions between alternative NAD(P)H dehydrogenases and AIF-like oxidoreductases

**Figure 4:**
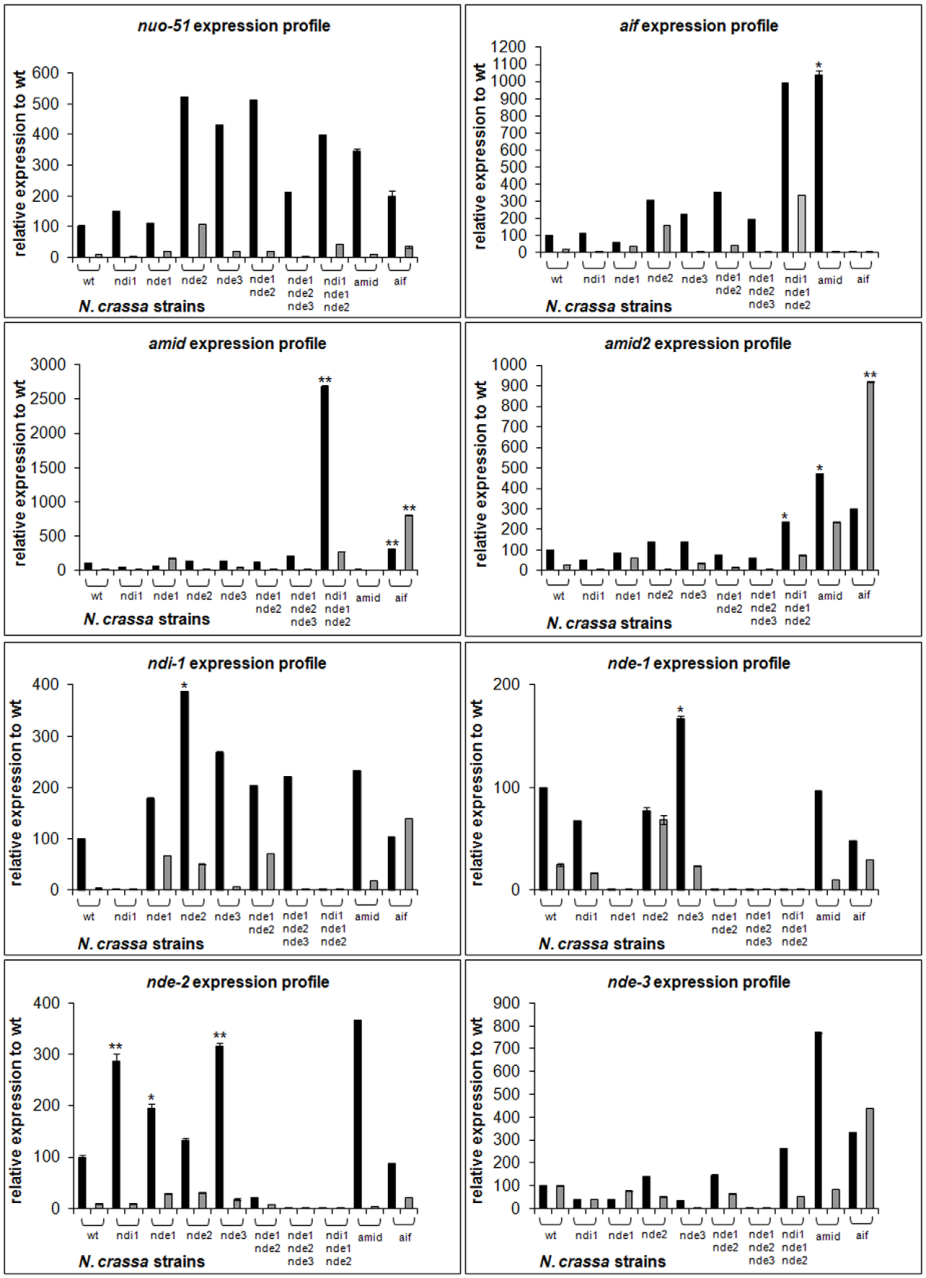
Expression profile of NAD(P)H oxidoreductases in *N. crassa* strains. Total RNA was prepared from wild type and mutant strains mycelia grown to early exponential phase (black, 12–16 h) or to late exponential phase (gray, 20–24 h). Expression of the indicated genes was quantified by RT-PCR using the primer combinations depicted in Table 2. Relative expressions were obtained per µg cDNA and are depicted as relative to wt. Data are expressed as the means ± S. D. of two independent experiments. Statistical significance was calculated with ANOVA followed by Tukey's Post Hoc test (*, *p*<0.05; **, *p*<0.01).

The mitochondrial respiratory chain of *N. crassa* has been characterized as highly branched with four alternative dehydrogenases bypassing the activity of complex I [11]. The identification of three more genes encoding putative oxidoreductases envisages significant genetic interactions between themselves and with the alternative dehydrogenases that may provide clues as to their function in Neurospora. Thus, we found it appropriate to assess the expression profile of alternative dehydrogenases and AIF-like genes in the different mutant strains (Fig. 4). We decided not to include amid2 in the expression profile study because, as stated before, it is a heterokaryotic strain with consequent heterogeneity in gene content. Each gene expression profile was evaluated in both early and late exponential phases of growth as we have previously demonstrated that expression of genes encoding mitochondrial respiratory enzymes varies widely depending on the stage of growth [36]. In addition, we have previously provided evidence that among mitochondrial NAD(P)H dehydrogenases, complex I (*nuo-51* transcript) appears to be the most abundant and that, with the exception of *nde-3*, genes encoding these enzymes are significantly downregulated from early to late exponential stage of growth [36], [40]. Herein, we show that AIF-like encoding genes are also downregulated from early to late exponential stage of growth, suggesting that the early exponential stage of growth in Neurospora is a period of soaring transcription events. Clearly, and corroborating the phylogenetic analysis depicted in Fig. 1B, the two classes of genes compensate among themselves. An analysis of the expression profiles in the various mutant strains provides evidence that there is an overall compensation concerning alternative dehydrogenases. Likewise, *aif*, *amid* and *amid-2* appear up regulated in each other mutant strains suggestive of overlapping roles and compensatory regulation mechanisms. Interestingly, expression of AIF-like encoding genes appears significantly up regulated in a triple mutant devoid of NDI1, NDE1 and NDE2 (Fig. 4), although we were not able to associate this increase with an obvious phenotype. Our most striking result concerns the expression of *nde-3*. Indeed, its expression does not appear to be regulated according to the remaining NAD(P)H dehydrogenases since we could not detect any expression variation in the respective respiratory mutants. In contrast, we observed a robust increase in the expression pattern of *nde-3* in aif and amid mutants, suggesting that NDE3 may be functionally redundant to AIF-like proteins. More so, these results once again corroborate the phylogenetic analysis depicting NDE3 as clustering with AIF and AMID proteins rather than with alternative NAD(P)H dehydrogenases (Fig. 1B).

## Discussion

AIF is nowadays regarded as a key player in both life and death exerting roles in a plethora of signal transduction pathways in the mitochondria and nucleus, respectively [41], [19], [21]. Within the last decade a number of AIF homologues, including AMID proteins, have been identified and characterized in various model organisms [42]. The fact that they present homology to NAD(P)H dehydrogenases with recognized function in the mitochondrial respiratory chain prompted us to search for AIF-like proteins in the Neurospora genome.

The *N. crassa* genome depicts three sequences presenting recognized homology to AIF and AMID proteins as well as to NAD(P)H oxidoreductases. The phylogenetic analysis revealed that the two families of enzymes separate into two distinct branches, encompassing members of either NAD(P)H dehydrogenases or of AIF-like proteins from a number of organisms, including *N. crassa* and *Homo sapiens*. Our most striking result concerns the clustering of NDE3 with AIF-like proteins rather than with NAD(P)H dehydrogenases. Interestingly, upon genetic profiling in several mutant strains, it was also clear that while genes encoding the remaining alternative dehydrogenases become up regulated under each other deficiencies, *nde-3* expression is relatively stable in the majority of respiratory mutant strains tested. Rather, we could detect a robust increase in *nde-3* transcript in the aif and amid mutant strains, suggesting a putative compensatory role for NDE3 yet uncharacterized. More so, we have previously characterized NDE3 as localized to both the mitochondria and the cytoplasm, although we have not yet dissected the functional relevance of this localization [36]. Given its similarity to AIF-like proteins we are tempted to speculate that NDE3 may play important roles in programmed cell death. Indeed, the alternative dehydrogenase NDI1 has already been described as the AMID homologue in *S. cerevisiae*, displaying similar apoptotic features of human AMID when over expressed [28].

In this study we established the localization of AIF and AMID proteins in Neurospora, and both AIF and AMID2 were found to display double localization patterns. Interestingly, both proteins were found in the cytoplasm and mitochondria of Neurospora, despite that only AIF depicts a recognized MLS signal. Moreover, although AIF was found mainly in the cytoplasm, the identification of AIF as a mitochondrial protein in our study corroborates recent reports by a Neurospora mitochondrial proteomic study [43]. However, we were unable to clearly determine its mitochondrial topology, and thus we cannot discard the possibility that AIF may just be attached to mitochondria. In the fungus *Podospora anserina* two AIFs have been reported, one localized to the mitochondria and another one to the cytoplasm [35]. Thus, it is conceivable that the single *N. crassa* AIF may have double localization to fulfill independent functions. As far as AMID proteins are concerned, much controversy has surrounded their localization. In fact, AMID has been described as being associated to the outer mitochondrial membrane [17] or localized to the cytoplasm [44]. In our study, AMID was found exclusively in the cytoplasm, consistent with more recent reports [25], whereas AMID2 was found to localize to both the mitochondria and the cytoplasm. Strikingly though, AMID2 was mostly undetectable in wild type extracts and appeared significantly up regulated in the amid mutant strain, suggesting overlapping functions of the two proteins. Indeed, gene expression profiling revealed that Neurospora *aif*, *amid* and *amid-2* transcripts become up regulated in each other mutant strains corroborating functional redundancy among the genes.

Previous reports have awarded AIF a specific role in the mitochondrial respiratory chain, specifically AIF deficiency was found to compromise oxidative phosphorylation by hindering complex I assembly and/or function and to a lower extent complex III [23]. Furthermore, both AIF and AMID proteins have been described as possessing oxidoreductase activity [22], [27]. Our results demonstrate that neither AIF nor AMID deficiency compromises mitochondrial respiratory activity in *N. crassa*. Indeed, respiration driven by cytosolic or matrix substrates did not differ significantly in either mutant when compared to the wild type strain, providing evidence that neither AIF nor AMID belong to the mitochondrial respiratory chain. More so, complex I assembly and supramolecular organization within the respiratory chain was similar in all strains.

In conclusion, we have provided evidence for the existence of AIF and AMID homologues in *N. crassa* that display characteristics of mammalian AIF-like proteins. However, AIF appears doubly localized to the mitochondria and cytoplasm, which may fulfill a fungal specific role. Furthermore, our results suggest that the alternative dehydrogenase NDE3 is a putative AIF-like gene. Unlike previous reports [23], [31], [35] disruption of AIF-like proteins in *N. crassa* does not affect the assembly and function of the mitochondrial respiratory chain.

Characterization of the roles played by *N. crassa* AIF and AMID homologues in programmed cell death will provide important insights regarding fungal specific mechanisms of cell death while laying the foundation for apoptosis research. More so, it will be extremely valuable to dissect the relationship between redox activity, respiratory chain and programmed cell death.

## Materials and Methods

### *N. crassa* strains and manipulations

Wild type *N. crassa* (FGSC#2489) and mutant strains aif (FGSC#11900), amid (FGSC#12090) and amid2 (FGSC#12511) were obtained from the Fungal Genetics Stock Center (FGSC) [45]. Strains with mutations in the respiratory chain dehydrogenase genes *ndi-1*, *nde-1*, *nde-2* and *nde-3* have been previously described [46]–[48], [36]. Double and triple mutants were obtained through genetic crosses between single mutants. General manipulation of Neurospora strains was performed according to standard procedures [49].

### Production of antibodies

The *aif* and *amid* full-length cDNAs and a 0.8 kb fragment of *amid-2* were amplified from the *N. crassa* M^−^ cDNA library by PCR (Polymerase chain reaction) using the specific primers: *aif* (5′–GAATTCGATGACATCCATGATTGCACTGC–3′ and 5′–GAATTCGTCATCAAAAA GGCAGGTGAATGG–3′); *amid* (5′– GGATCCGATGGGATCTATTGCCGTTG –3′ and 5′–GGATCCCGCCAATGCCTTCAAAGATG–3′); *amid-2* (5′–GGATCCAATGGCTTG CGACCTCAAGG–3′ and 5′–GGATCCCCGCACTGCCACTGTTTATAAG–3′). The PCR products were cloned in the pCR 2.1-TOPO vector (Invitrogen), digested with *Bam*HI (*amid* and *amid-2*) or *Eco*RI (*aif*) and subcloned into the pQE31 or pET28b expression vectors, respectively. The AIF, AMID and AMID2 proteins were thus expressed in *Escherichia coli* as fusion proteins containing an N terminal His-Tag. After purification the recombinant protein was used to generate rabbit polyclonal antisera [50]. Antisera against FKBP50 [51] and several subunits of complex I were used as previously described [52], [39].

### Preparation of total extracts, purified mitochondria and cytoplasm

*N. crassa* hyphae were homogenized with a grinding mill and the resulting suspension was saved as total extracts. The obtained homogenate was differentially centrifuged from which we obtained a pellet (crude mitochondria) and a supernatant fraction (cytoplasm), as previously described [53]. Mitochondria were purified by density gradient centrifugation upon layered on top of a step gradient made of 50% sucrose (7 ml), 40% sucrose (2 ml) and 30% sucrose (2 ml) in 10 mM Tris, pH 7.5 according to standard procedures (FGSC;http://www.fgsc.net/neurosporaprotocols/vm%20vacuole%20procedure.pdf). For fractionation of mitochondria a linear 30–60% (w/w) sucrose density gradient was subjected to centrifugation at 40 000×***g*** for 2 h. One-milliliter fractions were collected from the bottom (fraction 1) to the top (fraction 10).

### Oxygen consumption

*N. crassa* mitochondria were prepared as previously described [53]. Respiration was measured polarographically with a Clark-type oxygen electrode (Hansatech) at 25°C in a total volume of 1 ml. Mitochondrial assays contained 0.5–1 mg of protein, 0.3 M sucrose, 10 mM potassium phosphate (pH 7.2), 5 mM MgCl_2_, 1 mM EGTA, 10 mM KCl, 4 µM carbonyl cyanide m-chlorophenylhydrazone and 0.02% (w/v) BSA. The reactions were initiated by the addition of either 1 mM NADH or 1 mM NADPH. Internal respiratory activities were assayed in reaction medium containing 1 mM NAD^+^ and 5 mM pyruvate and were started upon addition of 10 mM malate.

Rotenone and antimycin were added to final concentrations of 20 µM and 0.2 µg/ml respectively. Integrity of mitochondria was assessed through the measurement of cytochrome c oxidase (EC 1.9.3.1) and malate dehydrogenase (EC 1.1.1.37) activities in the absence and presence of Triton X-100 [47].

### Blue Native-PAGE electrophoresis

Mitochondria from *N. crassa* strains were thawed on ice, centrifuged at 10 000 *g* for 5 min and the resulting pellet was suspended in solubilization buffer containing 50 mM NaCl, 50 mM imidazole/HCl (pH 7.0), 10% glycerol and 5 mM 6-aminocaproic acid. Mitochondria were solubilized with digitonin at a detergent/protein ratio of 4 g/g using a freshly prepared 10% detergent solution. The samples were centrifuged at 10 000 *g* for 30 min upon 30 min incubation on ice. Each lane was loaded with the mitochondrial extract containing 150 µg of protein prior to solubilization. For BN-PAGE (Blue Native- polyacrylamide gel electrophoresis), linear 4–13% gradient gels overlaid with a 3% stacking gel were used. Upon electrophoresis, gels were stained with either Coomassie blue or for NADH/NBT (Nitro-Blue Tetrazolium) activity, as previously described [54].

### Gene expression analysis by RT-PCR

Neurospora mycelium was grown to both early exponential (12–16 h growth, about 3 g/l) and late exponential phases (20–24 h growth, about 10 g/l) and total RNA was isolated with the Ilustra RNAspin Mini kit (GE Healthcare), quantified with the ND 1000 spectrophotometer (Nanodrop) and subsequently used to produce cDNA with SuperScript First-Strand Synthesis System kit (Invitrogen) according to the manufacturer instructions. RT-PCR (real time polymerase chain reaction) experiments were performed in an iCycler iQ5 with SYBR Green Supermix kit (Bio-Rad) using a 1/10 dilution of each cDNA, with annealing temperatures set at 60°C. Specific oligonucleotide primers (Table 2) were designed using the Beacon Designer program (PREMIER Biosoft International). Expression of the actin gene was measured in parallel assays to normalize the amount of cDNA per assay and relative expressions were obtained per µg cDNA. Two independent experiments were performed, each in triplicate.

**Table 2:** PCR oligonucleotide sequences.

Gene Product	*N. crassa* Accession Number	Oligonucleotide Sequence
*ndi-1*	NCU00153	F-GCCAACGCCAACGCCGATTCR-GTAGGCACCGCAAGCAATGACAAG
*nde-1*	NCU05225	F-GACGCCCGCCAGATCCGCAACAAGR-CCCGCCGCCGCAGACGACAAAG
*nde-2*	NCU11397	F-GGAGCCCATCCGCACCATTCTGR-CAGCACCGACACCAACCACCAG
*nde-3*	NCU09447	F-TCGTCATTCTCGGCGGCAACCR-CGGCAAGAGGTCAGGATGGATCAG
*amid*	NCU06061	F-TCAAGCGGTGCGGTGGTGCCATGR-CCTCGCCCGATGACACGCCGTTTG
*amid-2*	NCU12058	F-GAGCGACTGAGCGAGATCATCAAGR-CCCTCCACCACACTCCCATCC
*aif*	NCU05850	F-TCAACACCACGACGGGCGATATTGR-CCACCACCGACAACAACCACTCTG
*nuo-51*	NCU04044	F-TGAACGCCACCGCTGCCTACATCR-GCTGGTCTCCTCGCCGCACAC
*actin*	NCU04173	F-GGCATCACACCTTCTACAACGAGR-ATGTCAACACGGGCAATGGC

F-forward; R-reverse.

**Table 3:** Type II NAD(P)H:quinone oxidoreductases and AIF-like oxidoreductases used in the dendogram of Fig. 1B.

Abbreviation	*Protein ID in NCBI*	Species
*HsAIF*	NP_004199	*Homo sapiens*
*HsAMID*	AAM77596	*H. sapiens*
*MmAIF*	AAD16435	*Mus musculus*
*MmAMID*	CAM22220	*M. musculus*
*NcAIf*	XP_959841	*Neurospora crassa*
*NcAMID*	XP_959824	*N. crassa*
*NcAMID2*	XP_001728525	*N. crassa*
*NcNDI1*	XP_956666	*N. crassa*
*NcNDE1*	XP_961885	*N. crassa*
*NcNDE2*	XP_959008	*N. crassa*
*NcNDE3*	XP_958599	*N. crassa*
*PaAIF1*	XP_001906597	*Podospora anserina*
*PaAIF2*	XP_001903634	*P. anserina*
*PaAMID1*	XP_001905330	*P. anserina*
*PaAMID2*	XP_001905548	*P. anserina*
*PaPRG-3*	XP_001905340	*P. anserina*
*PaNDI1*	XP_001907841	*P. anserina*
*PaNDE1*	XP_001906747	*P. anserina*
*PaNDE2*	XP_001907894	*P. anserina*
*ScAIF*	NP_014472	*Saccharomyces cerevisiae*
*ScNDI1*	NP_013586	*S. cerevisiae*
*ScNDE1*	NP_013865	*S. cerevisiae*
*ScNDE2*	NP_010198	*S. cerevisiae*
*YlNDE1*	XP_503592	*Yarrowia lipolytica*
*YlNDE2*	XP_505856	*Y. lipolytica*

ID-identification; NCBI-National Center for Biotechnology Information.

### Miscellaneous

The following techniques were performed according to standard protocols: PCR and general cloning procedures [55], protein determination [56], SDS-PAGE [57] and Western blot [58].

### Statistical analysis

Statistical analyses were performed using One-way ANOVA followed by Tukey's Post Hoc test. Data are presented as the means ± S.D. of at least two independent experiments. A *p* value<0.05 was considered significant.

Independent-Samples T test was used for comparison of two means. p<0.05 (*), p<0.01(**).
